# What determines medical students’ career preference for general practice residency training?: a multicenter survey in Japan

**DOI:** 10.1186/s12930-018-0039-9

**Published:** 2018-01-27

**Authors:** Kenya Ie, Akiko Murata, Masao Tahara, Manabu Komiyama, Shuhei Ichikawa, Yousuke C. Takemura, Hirotaka Onishi

**Affiliations:** 1 Department of General Internal Medicine, Kawasaki Municipal Tama Hospital/St. Marianna University School of Medicine, 1-30-37 Shukugawara, Kawasaki, Kanagawa 214-8525 Japan; 2 Department of Family Medicine, Mie University School of Medicine, 2-174 Edobashi, Tsu-shi, Mie 514-8507 Japan; 3 Family Practice Center of Okayama, 292-1 Toyosawa, Katsuta-gun, Nagi-cho, Okayama 708-1323 Japan; 4 Iwakura Station Tahara Clinic, 291-1 Chuzaiji, Iwakura, Sakyo-ku, Kyoto-shi, Kyoto 606-0021 Japan; 5 Thank You All, Family Clinic Hiratsuka, 215-3 Okazaki, Hiratsuka-shi, Kanagawa 259-1212 Japan; 6 Department of Education and Research in Family and Community Medicine, Mie University Graduate School of Medicine, 2-174 Edobashi, Tsu-shi, Mie 514-8507 Japan; 7 International Research Center for Medical Education, Graduate School of Medicine, The University of Tokyo, 7-3-1, Hongo, Bunkyo-ku, Tokyo, 113-0033 Japan

**Keywords:** General practice, Primary care, Family practice, Career choice, Medical students, Surveys and questionnaires

## Abstract

**Background:**

Few studies have systematically explored factors affecting medical students’ general practice career choice. We conducted a nationwide multicenter survey (Japan MEdical Career of Students: JMECS) to examine factors associated with students’ general practice career aspirations in Japan, where it has been decided that general practice will be officially acknowledged as a new discipline.

**Methods:**

From April to December 2015, we distributed a 21-item questionnaire to final year medical students in 17 medical schools. The survey asked students about their top three career preferences from 19 specialty fields, their demographics and their career priorities. Multivariable logistic regression was used to determine the effect of each item.

**Results:**

A total of 1264 responses were included in the analyses. The top three specialty choice were internal medicine: 833 (65.9%), general practice: 408 (32.3%), and pediatrics: 372 (29.4%). Among demographic factors, “plan to inherit other’s practice” positively associated with choosing general practice, whereas “having physician parent” had negative correlation. After controlling for potential confounders, students who ranked the following items as highly important were more likely to choose general practice: “clinical diagnostic reasoning (adjusted odds ratio (aOR): 1.65, 95% CI 1.40–1.94)”, “community-oriented practice (aOR: 1.33, 95% CI 1.13–1.57)”, and” involvement in preventive medicine (aOR: 1.18, 95% CI 1.01–1.38)”. On the contrary, “acute care rather than chronic care”, “mastering advanced procedures”, and “depth rather than breadth of practice” were less likely to be associated with general practice aspiration.

**Conclusions:**

Our nationwide multicenter survey found several features associated with general practice career aspirations: clinical diagnostic reasoning; community-oriented practice; and preventive medicine. These results can be fundamental to future research and the development of recruitment strategies.

## Background

Family medicine training in Asian countries has been developed mostly in partnership with other western countries since the 1970s. Commonwealth member countries like Singapore and Malaysia have started family medicine training several decades ago, whereas other nations have established it more recently [1].

Japanese physicians have been board certified by the individual society of their specialty. Although ex-specialists have played a major role in Japanese primary care, there was no formal family medicine training until recently [11]. Starting in 2018, however, a new board certificate system for physicians’ specialties by a third-party organization will be implemented. Along with this reform, it has been decided that general practice, including family medicine, will be officially acknowledged as a new basic discipline. Based on such historical context, it is anticipated that the number of applicants for general practice will increase among medical students and interns.

In Europe and North America, accumulating evidence has elucidated factors associated with choosing family medicine and general practice as a career among medical students and junior doctors. Within the existing literature on medical career choice, several demographic factors have been shown to positively influence the choice of general practice/family medicine: being female [2–5], coming from rural background [2, 5, 6], having no family members in the healthcare field [6, 7]. In addition to the demographic factors, career intentions such as interest in broad scope practice [7–9], interest in community-oriented practice [10, 11], work-life balance [11–13], and societal orientation [6–8] have consistently predicted general practice/family medicine career choice.

On the contrary, few studies have systematically explored factors affecting specialty choice of Japanese medical students and junior doctors, especially in general practice/family medicine. Our previous qualitative study revealed a range of factors affecting the choice of family medicine as their specialty including but not limited to “community/rural orientedness” and “multifaceted orientation” [11]. More recently, a cross-sectional study at a Japanese university revealed that admission from hometown, intent for rural practice, and work-life balance were positive influences, while presence of medical relatives and scientific orientation were negatively associated with general practice career choice [14]. To our knowledge, however, no multicenter study has been conducted with a focus on general practice aspiration under the new board certificate system.

The purpose of this study was to conduct a nationwide multicenter survey (Japan MEdical Career of Students: JMECS) to examine factors associated with general practice career aspirations among Japanese medical students.

## Methods

### Study population

From April to December 2015, 17 medical schools (14 public schools and three private schools) which were selected by convenience sampling and gave permission for study participation were included in the survey. We distributed a 21-item questionnaire to medical students who had enrolled in their final year at the time of April 2015 in the 17 medical schools. We were able to sample approximately 21% of 81 medical schools in Japan in 2015.

### Measurement

The items included in the questionnaire were determined based on a literature review [2–14], including our previous qualitative study results, and discussion among the study team. Given the paucity of existing evidence in a Japanese context, we included items thought to reflect the uniqueness of Japanese general practice. The survey asked students to select top three career options from 19 newly determined specialty fields as an outcome variable: internal medicine, pediatrics, dermatology, psychiatry, surgery, orthopedics, obstetrics and gynecology, ophthalmology, otorhinolaryngology, urology, neurosurgery, radiology, anesthesiology, pathology, clinical laboratory, emergency medicine, plastic surgery, rehabilitation, and general practice. The other explanatory variables included participant demographics (e.g. age, sex) and 14 questions regarding their career intention, using 6-point Likert scales ranging from 1 (strongly disagree) to 6 (strongly agree). All the data collected from medical students were anonymous except for the name of the medical school. Before the data were handed over to the researchers, the school names were coded and de-identified by an honest broker using a linking file.

### Statistical analysis

The outcome variable was dichotomized into two categories based on whether general practice was included in up to three choices or not. Among explanatory variables, “sex”, “job or other field experiences prior to medical school”, “having physician parent”, and “plan to inherit other’s practice” were treated as binary; “birthplace” was treated as nominal, and “age” was treated as a continuous variable. The responses to the Likert scale of 14 career intention questions were treated as continuous, assuming that the variables have interval properties. Univariable and multivariable logistic regression were used to examine the effect of each demographic factor and career intentions in terms of odds ratio and 95% confidence intervals. We considered p values less than or equal to 0.05 as significant. STATA SE14.0 was used for all analyses. Since the generation of parsimonious model was not our primary objective, only the saturated model was fitted in our logistic regression analyses. Hosmer–Lemeshow Goodness of Fit test was applied to test the overall model fit. Multicollinearity was tested based on variance inflation factor with a cutoff of 10. Model discrimination was tested using c-statistics.

The study protocol was approved by the Institutional Ethical Committee of Mie University Graduate School of Medicine (No. 1482).

## Results

A total of 1408 responses out of a possible 1903 medical students (74.0%) in the 17 medical schools were obtained. Among them, 1264 responses were included in the final analyses after removing invalid or insufficient responses. Distribution of the participants’ sex, age, birth place, job or other field experiences prior to medical school, physician parent, plan to inherit other’s practice, and career priorities are shown in Table 1. The survey participants had a median age of 24, 66.3% were male, 32.1% had a physician parent, and 11.3% had a positive intent to inherit an existing practice. Among the students with an intention to inherit, 92.3% had a physician parent. On the contrary, only 32.5% of participants with a physician parent revealed their intention to inherit. The top five specialty fields chosen by students and their numbers were internal medicine 833 (65.9%), general practice 408 (32.3%), pediatrics 372 (29.4%), surgery 344 (27.2%), and emergency medicine 244 (19.3%).

**Table 1:** Characteristics of GP candidates and non-GP candidates among Japanese medical student

	Total (N = 1264)	GP (N = 405)	Non-GP (N = 846)
Demographics; no. (%) of students			
Age, median (range), years	24 (23–58)	24 (23–58)	24 (23–52)
Sex (male)	838 (66.3)	274 (67.2)	564 (65.9)
Hometown			
Urban	267 (21.1)	72 (17.7)	195 (22.8)
Relatively urban	287 (22.7)	102 (25)	185 (21.6)
Relatively rural	401 (31.7)	130 (31.9)	271 (31.7)
Rural	309 (24.5)	104 (25.5)	205 (24)
Other academic or professional experiences prior to medical school	286 (22.6)	102 (25)	184 (21.5)
Physician parent	406 (32.1)	114 (27.9)	292 (34.1)
Intent to inherit existing practice	143 (11.3)	55 (13.5)	88 (10.3)
Career priorities^a^; mean (SD)			
Mastering advanced procedures	4.83 (1.00)	4.60 (1.03)	4.93 (0.97)
Work life balance	4.89 (0.93)	4.90 (0.89)	4.89 (0.95)
Frequent patient communication	4.82 (0.89)	5.01 (0.82)	4.73 (0.91)
Opening own clinic	3.33 (1.35)	3.48 (1.34)	3.26 (1.35)
Involvement in preventive medicine	4.06 (1.13)	4.40 (1.04)	3.90 (1.14)
Involvement in terminal care	3.77 (1.15)	4.06 (1.03)	3.63 (1.17)
Acute care rather than chronic care	4.11 (1.06)	3.96 (1.02)	4.18 (1.07)
Not treat patients with psychosocial problems	2.75 (1.19)	2.50 (1.14)	2.87 (1.19)
Income	4.17 (1.00)	4.04 (1.08)	4.23 (0.96)
Access to advanced medical fields	4.28 (0.98)	4.06 (0.97)	4.39 (0.96)
Clinical diagnostic reasoning	4.31 (1.00)	4.60 (0.95)	4.17 (0.99)
Depth rather than breadth of practice	3.97 (1.02)	3.64 (0.97)	4.13 (1.01)
Involvement in global health	3.37 (1.13)	3.45 (1.13)	3.32 (1.12)
Community-oriented practice	4.09 (1.05)	4.47 (0.98)	3.91 (1.03)

^a^Please select one of the following options which best describes your thoughts regarding your career priorities.” (1 = strongly disagree, 6 = strongly agree)

Univariate analysis found those born in suburban areas compared to urban areas, and not having a physician parent, both had significant effects on students’ general practice career preference (Table 2). However, after adjusting for other covariates in the multivariable logistic regression, “having a physician parent” and “intent to inherit existing practice” had a negative association with choosing general practice among the medical students demographics (Table 2).

**Table 2:** Crude and adjusted odds ratio of characteristics of GP candidates among Japanese medical students (N = 1264)

OR (95% CI)	Crude	Adjusted
Demographics		
Sex (male)	0.94 (0.74, 1.21)	0.85 (0.64, 1.15)
Hometown		
Urban	–	–
Relatively urban	1.49* (1.04, 2.15)	1.45 (0.96, 2.17)
Relatively rural	1.30 (0.92, 1.83)	1.24 (0.85, 1.83)
Rural	1.37 (0.96, 1.97)	1.25 (0.84, 1.88)
Other academic or professional experiences prior to medical school	1.22 (0.92, 1.61)	1.23 (0.90, 1.68)
Physician parent	0.75* (0.58, 0.96)	0.59* (0.42, 0.83)
Intent to inherit existing practice	1.36 (0.95, 1.95)	1.64* (1.01, 2.68)
Career priorities		
Mastering advanced procedures	0.72* (0.64, 0.81)	0.75* (0.65, 0.87)
Work life balance	1.02 (0.90, 1.15)	0.89 (0.76, 1.06)
Frequent patient communication	1.47* (1.28, 1.70)	1.17 (0.97, 1.42)
Opening own clinic	1.13* (1.03, 1.23)	1.08 (0.95, 1.22)
Involvement in preventive medicine	1.51* (1.35, 1.70)	1.18* (1.01, 1.38)
Involvement in terminal care	1.41 (1.26, 1.58)	1.10 (0.95, 1.28)
Acute care rather than chronic care	0.82* (0.73, 0.92)	0.85* (0.74, 0.98)
Not treat patients with psychosocial problems	0.76* (0.68, 0.84)	0.91 (0.80, 1.04)
Income	0.83* (0.73, 0.93)	0.92 (0.79, 1.08)
Access to advanced medical fields	0.70* (0.62, 0.80)	0.85 (0.71, 1.02)
Clinical diagnostic reasoning	1.60* (1.40, 1.82)	1.65* (1.40, 1.94)
Depth rather than breadth of practice	0.61* (0.54, 0.69)	0.69* (0.59, 0.81)
Involvement in global health	1.11* (1.00, 1.23)	1.03 (0.90, 1.18)
Community-oriented practice	1.77* (1.55, 2.01)	1.33* (1.13, 1.57)

Dependent variable: whether general practice was included in up to three choices (1) or not (0)

Adjusted pseudo R^2^ = 0.1572; AIC = 1.093329

* Statistically significant at an alpha level of 0.05

As for the 14 career priorities, seven positive influences and six negative influencers were identified in crude (or univariate) analyses (Table 2). After adjusting for other covariates in the multivariable logistic regression, three positive influencers and three negative influencers remained significant. Medical students who ranked “clinical diagnostic reasoning”, “community-oriented practice”, and “involvement in preventive medicine” as highly important were more likely to choose general practice, whereas “acute care rather than chronic care”, “mastering advanced procedures”, and “depth rather than breadth of practice” were less likely to be associated with general practice aspiration (Table 2).

## Discussion

The survey participants revealed a similar distribution of age and gender to a national sample of recent medical school graduates in Japan [15]. Among the study participants, general practice was selected as one of the career options by approximately 32% of medical students in their final year. “Clinical diagnostic reasoning”, “community-oriented practice”, and “involvement in preventive medicine” were positively associated with the selection of general practice. A student’s community orientation has been shown to predict primary care career preference and was a factor affecting family medicine career choice among Japanese physicians [11]. On the contrary, the ranking of “acute care rather than chronic care”, “mastering advanced procedures”, and “depth rather than breadth of practice” as of higher importance were less likely to be associated with an aspiration for general practice aspiration. This has been described in other studies [13].

Of 14 career priorities, “clinical diagnostic reasoning” was the strongest predictor of general practice choice, a finding not common in previous literatures. In many Japanese medical schools, the department of general practice or family medicine has been in charge of teaching fundamental clinical reasoning skills to medical students. Such context could have contributed to the strong positive association between clinical diagnostic reasoning and general practice choice among study participants.

Two demographic factors, “having physician parent” and “plan to inherit other’s practice”, were found to have significant association with choosing general practice, even after controlling for other covariates. Having physician parent was negatively associated with general practice choice, which is concordant with existing literature [6, 7] from North America and Europe. One potential reason is that the perceived prestige of other medical specialties among physician parents could negatively influence their children’s general practice choice. On the other hand, having family or friends in general practice was found to have positive association with general practice career choice in a study [15]. Thus, it is possible that the effect of family physicians may vary according to the specialty of parents. A positive association between the intention to inherit existing practice and general practice choice would have reflected students’ perceived usefulness of general practice residency to develop expertise as a future private practice owner.

Some previously known demographic factors, such as being female [2–5] and coming from rural background [2, 5, 6], had no association between students’ general practice choice. In our study, participants were asked to select the degree of rurality of their birthplace based on subjective judgment. This could be attributed to the heterogeneity of birthplace classification, which potentially could have led to a null result. In addition, the different definition of rural areas among countries should be taken into consideration for future research.

The role of gender in career choice has been well studied and is known to be affected by nationality. A qualitative study explored Japanese female doctors’ perspectives on specialty choice and found that they tend to make a choice based on conventional gender roles where women tend to spend more time on household duties [16]. It is possible that general practice, which is not well-established yet in Japan, could have been perceived as too unpredictable and insecure a career path. Such insecurities of general practice career could have been perceived as an unreasonable choice for female medical students. While family medicine preference at entry to medical school is known to increase the likelihood of choosing a family medicine career [7, 8], this item was not included in our study as we assumed that a general practice career path had not been widely available at the time of study participants’ medical school entry.

Our results reflect how general practice is perceived by medical students, and they are a little different from that in other countries where general practice/family medicine is well-established. Further studies are required to confirm factors affecting students’ career choice to determine strategies to facilitate general practice career choice in Japan.

### Limitations

Despite the thorough development of the questionnaire and relatively large sample size, our study has several limitations. First of all, our outcome measurement was career aspiration during the final year of medical school. Thus, the actual enrolment in general practice residency and subsequent retention rate should also be considered in the future. Secondly, due to convenience sampling of medical schools, the representativeness of our study results to the general medical student population would be limited. In addition, the study results may not be generalizable to other countries. To our knowledge, however, this is the first and largest nationwide survey conducted across multiple Japanese medical schools.

## Conclusions

Our nationwide multicenter survey found several important factors associated with general practice career aspirations among Japanese medical students. Clinical diagnostic reasoning, community-oriented practice, and preventive medicine were three major career priorities among students selected general practice. These results can be fundamental to future research and the development of recruitment strategies.

## Abbreviations

JMECS: Japan MEdical Career of Students
OR: odds ratio
